# Supplementary material for: Species traits and network structure predict the success and impacts of pollinator invasions
Source: Nat Commun. 2018 May 31;9:2153. doi: 10.1038/s41467-018-04593-y (PMC5981428; doi:10.1038/s41467-018-04593-y)
Supplement: Supplementary file 1 — Supplementary Information [file 41467_2018_4593_MOESM1_ESM.pdf]

Supporting Information for

Species traits and network structure predict the success and impacts of pollinator invasions

Fernanda S. Valdovinos*, Eric L. Berlow, Pablo Moisset de Espanés, Rodrigo Ramos-Jiliberto, Diego P. Vázquez & Neo D. Martinez

*Correspondence to: fsvaldov@umich.edu

Supplementary Information includes:

Supplementary Fig. 1. Alien abundance increases with aliens' number of interactions more in low than high mortality scenarios for efficient but not for average foragers.

Supplementary Table 1. Model parameters and state-variables.

Supplementary Table 2. CART analysis results of the effect of efficient alien foragers on natives for all data.

Supplementary Table 3. CART analysis results of the effect of efficient alien foragers on natives for pollinators' high mortality rate

Supplementary Table 4. Full description and results of our Generalized Linear Models.

Supplementary Fig. 1. Alien abundance increases with aliens' number of interactions more in low than high mortality scenarios for efficient but not for average foragers.

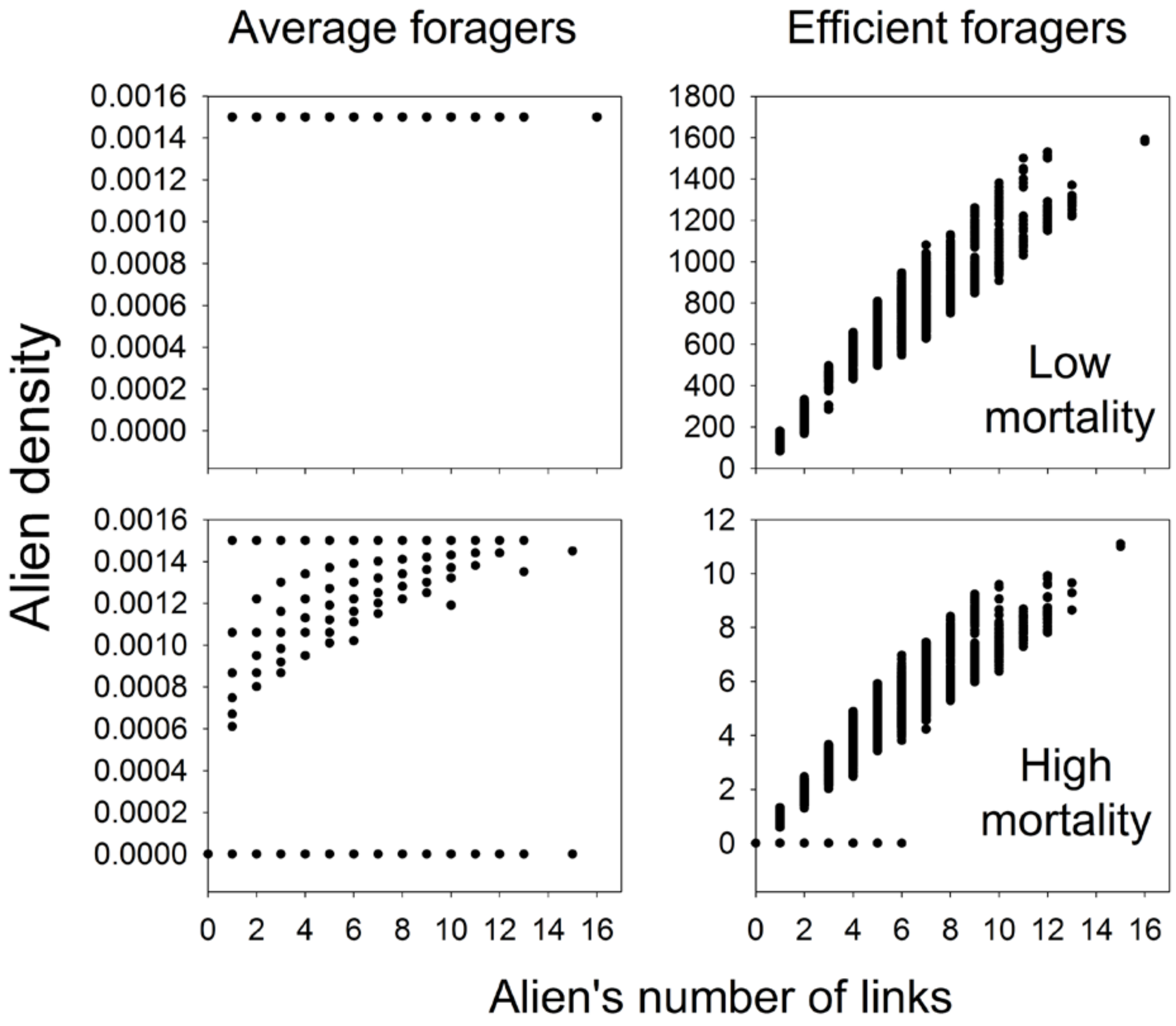

Supplementary Table 1. Model parameters and state-variables

Definition	Symbol	Dimension	Mean value
Density of plant population i	p_i	individuals area^{-1}	0.5*
Density of animal population j	a_j	individuals area^{-1}	0.5*
Total density of floral resources of plant population i	R_i	mass area^{-1}	0.5*
Foraging effort	α_{ij}	None	1 /k_{aj}*
Visitation efficiency	τ_{ij}	visits area time^{-1} individuals^{-1} individuals^{-1}	1
Expected number of seeds produced by a pollination event	e_{ij}	individuals visits^{-1}	0.8
Per capita mortality rate of plants	μ_i^P	time^{-1}	0.001
Conversion efficiency of floral resources to pollinator births	c_{ij}	individuals mass^{-1}	0.2
Per capita mortality rate of pollinators	μ_j^A	time^{-1}	0.05/ 0.001*
Pollinator extraction efficiency of resource in each visit (linear model)	b_{ij}	individuals visits^{-1}	0.4
Maximum fraction of total seeds that recruit to plants	g_i	None	0.4
Inter-specific competition coefficient of plants	u_i	area individuals^{-1}	0.06
Intra-specific competition coefficient of plants	w_i	area individuals^{-1}	1.2
Production rate of floral resources	βi	mass individuals^{-1} time^{-1}	0.2
Self-limitation parameter of resource production	ϕ_{ij}	time^{-1}	0.04
Adaptation rate of foraging efforts of pollinators	G_j	None	2

Values were drawn from a uniform random distribution with the specified mean, and variances of 10% and 0% of means for plants' and animals' parameters, respectively. Asterisks indicate initial conditions, k_{aj} is the number of interactions of animal j and the two values of animal mortality rate used, the highest for animal introductions and the lowest for plant introductions.

Supplementary Table 2. Classification and Regression Tree (CART, n=21,600) analysis of the density and effects on natives of 'efficient foragers' as a function of 30 properties of networks and alien pollinator species.

	Alien density	Pollinators' persistence	Pollinators' density	Plants' density	Total floral resources	Resource consumption by pollinators
**5-folded R^2**	0.99	0.89	0.99	0.75	0.93	0.89
**Main Contributors**	Pollinators' mortality: 61%<br>Fraction of plant sp visited by the alien: 37%	Alien's mean Jaccardian index: 62%<br>Connectivity of the most generalist plant sp: 13%<br>Fraction of plant sp visited by the alien: 11%	Fraction of plant sp visited by the alien: 99%	Alien density: 68%<br>Fraction of specialist native pollinators: 16%	Fraction of plant sp visited by the alien: 99%	Alien's mean Jaccardian index: 61%<br>Connectivity of the most generalist plant sp: 13%<br>Fraction of plant sp visited by the alien: 12%

Separated analyses were conducted for each of the 6 continuous outcomes (i.e., density of the aliens and their effects on the persistence and density of native pollinators, the density and total floral resources of native plants, and the resource consumption by native pollinators). Alien density was added to the CART analyses of the 5 types of aliens' effects on natives. Main contributors displayed include only factors that account for >5% of the variance within the 21,600 introductions of efficient foragers.

Supplementary Table 3. Classification and Regression Tree (CART, n=10,800) analysis of the density and effects on natives of ‘efficient foragers’ in a ‘high mortality scenario’ as a function of 30 properties of networks and alien pollinator species.

	Alien density	Pollinators’ persistence	Pollinators’ density	Plants’ density	Total floral resources	Resource consumption by pollinators
**5-folded R^2**	0.97	0.85	0.98	0.82	0.93	0.85
**Main Contributors**	Alien’s # links: 94%	Alien’s mean Jaccardian index: 63%<br>Connectivity of the most generalist plant sp: 13%<br>Fraction of plant sp visited by the alien: 12%	Fraction of plant sp visited by the alien: 99%	Alien density: 60%<br>Fraction of specialist native pollinators: 19%	Fraction of plant sp visited by the alien: 99%	Alien’s mean Jaccardian index: 63%<br>Connectivity of the most generalist plant sp: 13%<br>Fraction of plant sp visited by the alien: 12%

Separated analyses were conducted for each of the 6 continuous outcomes (i.e., density of the aliens and their effects on the persistence and density of native pollinators, the density and total floral resources of native plants, and the resource consumption by native pollinators). Alien density was added to the CART analyses of the 5 types of aliens’ effects on natives. Main contributors displayed include only factors that account for >5% of the variance within the 10,800 introductions of efficient foragers for high pollinators’ mortality.

Supplementary Table 4. Generalized Linear Models predicting the effect of alien pollinators on native species

	Estimate	Std. Error	t-value	Pr(>|t|)	
Alien effect on pollinators' persistence (AIC: -5153.8, Null/ Residual deviance: 4.89/ 0.93 on 1199/1185 degrees of freedom. Dispersion parameter for gaussian family: 0.00079)					
(Intercept)	-0.11	0.03	-3.40	0.0006	***
Alien foraging efficiency	0.04	0.02	2.46	0.014	*
Alien mean Jaccardian index	1.52	0.45	3.37	2 e-14	***
Connectivity of most generalist plant sp	0.014	0.003	4.56	5 e-6	***
Fraction of plant sp visited by alien	0.10	0.07	1.5	0.13	
Alien foraging efficiency * Alien mean Jaccardian	-1.14	0.25	-4.63	4e-6	***
Alien foraging efficiency * Connectivity of most generalist plant sp	-0.007	0.002	-4.31	2 e-5	***
Alien foraging efficiency * Fraction of plant sp visited by alien	0.20	0.03	6.56	8 e-11	***
Alien mean Jaccardian * Connectivity of most generalist plant sp	-0.14	0.05	-2.64	0.009	**
Alien mean Jaccardian * Fraction of plant sp visited by alien	-0.75	1.02	-0.73	0.46	
Connectivity of most generalist plant sp * Fraction of plant sp visited by alien	-0.036	0.006	-6.27	5 e-10	***
Alien foraging efficiency * Alien mean Jaccardian * Connectivity of most generalist plant sp	0.10	0.03	3.43	0.0006	***
Alien foraging efficiency * Alien mean Jaccardian * Fraction of plant sp visited by alien	-0.88	0.54	-1.63	0.1	
Alien mean Jaccardian * Connectivity of most generalist plant sp * Fraction of plant sp visited by alien	0.26	0.12	2.18	0.029	*
Interaction among 4 predictors	-0.07	0.06	-1.18	0.2	
Alien effect on native pollinator density (AIC: -11619, Null/ Residual deviance: 0.027 / 0.0043 on 1199/ 1185 degrees of freedom, Dispersion parameter for gaussian family: 3.6e-06)					
(Intercept)	-3 e-3	2 e-3	-1.51	0.1	
Alien foraging efficiency	-0.6 e-3	1 e-3	-0.52	0.6	
Alien mean Jaccardian index	2 e-2	3 e-2	0.69	0.5	
Connectivity of most generalist plant sp	-3 e-4	2 e-4	1.67	0.1	

Fraction of plant sp visited by alien	-9.5 e-3	4.7 e-3	-2.03	0.04	*
Alien foraging efficiency * Alien mean Jaccardian	3 e-4	1.7 e-2	0.02	1	
Alien foraging efficiency * Connectivity of most generalist plant sp	1 e-4	1 e-4	0.61	0.5	
Alien foraging efficiency * Fraction of plant sp visited by alien	2.6 e-2	0.2 e-2	12.9	< 2 e-16	***
Alien mean Jaccardian * Connectivity of most generalist plant sp	-3 e-3	4 e-3	-0.7	0.48	
Alien mean Jaccardian * Fraction of plant sp visited by alien	-1.1 e-1	0.7 e-2	-1.6	0.1	
Connectivity of most generalist plant sp * Fraction of plant sp visited by alien	-2 e-3	0.4 e-3	-5.2	2 e-7	***
Alien foraging efficiency * Alien mean Jaccardian * Connectivity of most generalist plant sp	0.3 e-4	2 e-3	0.15	0.9	
Alien foraging efficiency * Alien mean Jaccardian * Fraction of plant sp visited by alien	1.9 e-2	3.6 e-2	0.5	0.6	
Alien mean Jaccardian * Connectivity of most generalist plant sp * Fraction of plant sp visited by alien	1.5 e-2	0.8 e-2	1.8	0.07	
Interaction among 4 predictors	-4 e-3	4 e-3	-0.96	0.34	
Alien effect on native plant density (AIC: -14201, Null/ Residual deviance: 0.0011 / 0.0005 on 1199/1192 degrees of freedom. Dispersion parameter for gaussian family: 4.2 e-07)					
(Intercept)	-3 e-3	6 e-4	-4.57	5 e-06	***
Alien foraging efficiency	3 e-3	3 e-4	8.20	6 e-16	***
Alien density	-2 e-4	4 e-1	0.00	1.00	
Fraction of specialist native pollinator sp	2 e-3	7 e-4	3.42	0.0006	***
Alien foraging efficiency * Alien density	-1 e-4	2 e-1	-0.001	1.00	
Alien foraging efficiency * Fr. of specialist native pollinator sp	-2 e-3	5 e-4	-4.65	4 e-6	***
Interaction among 3 predictors	-2 e-7	6 e-5	-0.003	1.00	

Models were fit on data from independent introduction trials over 1200 networks (600 each for average and efficient alien foragers, consisting of 100 nested and 100 non-nested networks for each richness/connectance combination: 40/0.3, 90/0.15, 200/0.06 here tested, see Methods in main text). Only the best model (based on AIC) is shown for each effect. Significance codes for P-values: 0 ‘***’, 0.001 ‘**’, 0.01 ‘*’.
